# Dose calculation of Acuros XB and Anisotropic Analytical Algorithm in lung stereotactic body radiotherapy treatment with flattening filter free beams and the potential role of calculation grid size

**DOI:** 10.1186/s13014-015-0357-0

**Published:** 2015-02-26

**Authors:** Baotian Huang, Lili Wu, Peixian Lin, Chuangzhen Chen

**Affiliations:** Department of Radiation Oncology, Cancer Hospital of Shantou University Medical College, 7 Raoping Road, Shantou, 515031 China; Department of Nosocomial Infection Management, The Second Affiliated Hospital of Shantou University Medical College, 69 North Dongxia Road, Shantou, 515041 China

**Keywords:** Dose difference, Acuros XB, Anisotropic analytical algorithm, Stereotactic body radiotherapy, Calculation grid size

## Abstract

**Background:**

The study aimed to appraise the dose differences between Acuros XB (AXB) and Anisotropic Analytical Algorithm (AAA) in stereotactic body radiotherapy (SBRT) treatment for lung cancer with flattening filter free (FFF) beams. Additionally, the potential role of the calculation grid size (CGS) on the dose differences between the two algorithms was also investigated.

**Methods:**

SBRT plans with 6X and 10X FFF beams produced from the CT scan data of 10 patients suffering from stage I lung cancer were enrolled in this study. Clinically acceptable treatment plans with AAA were recalculated using AXB with the same monitor units (MU) and identical multileaf collimator (MLC) settings. Furthermore, different CGS (2.5 mm and 1 mm) in the two algorithms was also employed to investigate their dosimetric impact. Dose to planning target volumes (PTV) and organs at risk (OARs) between the two algorithms were compared. PTV was separated into PTV_soft (density in soft-tissue range) and PTV_lung (density in lung range) for comparison.

**Results:**

The dose to PTV_lung predicted by AXB was found to be 1.33 ± 1.12% (6XFFF beam with 2.5 mm CGS), 2.33 ± 1.37% (6XFFF beam with 1 mm CGS), 2.81 ± 2.33% (10XFFF beam with 2.5 mm CGS) and 3.34 ± 1.76% (10XFFF beam with 1 mm CGS) lower compared with that by AAA, respectively. However, the dose directed to PTV_soft was comparable. For OARs, AXB predicted a slightly lower dose to the aorta, chest wall, spinal cord and esophagus, regardless of whether the 6XFFF or 10XFFF beam was utilized. Exceptionally, dose to the ipsilateral lung was significantly higher with AXB.

**Conclusions:**

AXB principally predicts lower dose to PTV_lung compared to AAA and the CGS contributes to the relative dose difference between the two algorithms.

## Introduction

The Anisotropic Analytical Algorithm (AAA), a convolution/superposition method, is widely utilized for dose calculation in Eclipse treatment planning system [1,2]. However, AAA was reported to significantly overestimate the dose near air-tissue interfaces [3]. Recently, a new dose calculation algorithm named Acuros XB (AXB) has been implemented for clinical use. This new algorithm explicitly solves the linear Boltzmann transport equation that describes the macroscopic behavior of radiation particles as they travel through and interact with matters [4]. AXB was found to provide good agreement with Monte Carlo (MC) algorithm [5-7] and achieved more accurate dose prediction than AAA in heterogeneous phantom [2,6-10].

The dose difference between the two algorithms has became an issue of concern in the past few years and several publications have investigated the dosimetric impact of AXB in breast cancer, non-small cell lung cancer (NSCLC) and nasopharyngeal carcinoma with AAA as a benchmark [4,11,12]. The dose difference between the two algorithms was reported to be closely related to the beam energy, field size and the density of the materials [6]. However, they failed to consider the potential impact of the calculation grid size (CGS), which was reported to be associated with dose variation [13,14]. So far, the dose difference between AAA and AXB due to the use of different CGS in lung stereotactic body radiotherapy (SBRT) treatment remains unknown and the effect of it requires further investigation.

On the other hand, although two studies have evaluated the dosimetric impact of AXB in SBRT plan for lung cancer [15,16], little information has been focused on the dose difference generated from flattening filter free (FFF) beams, which was widely used in lung SBRT treatment for its improved treatment efficiency [17,18]. Because FFF beams provide softer spectrum than flattened beams [19], investigation of their impact is also essential.

Therefore, the aim of this study is to quantitatively determine the dose differences between AXB and AAA in SBRT treatment for lung cancer with FFF beams. Furthermore, the potential role of the CGS on the dose differences between the two algorithms was also discussed.

## Methods

### Beam configuration

The same set of beam data (including percentage depth dose curve, profiles and output factors) used by AAA and measured in a three-dimensional water scanning system (PTW, Germany) for field sizes from 3 × 3 to 40 × 40 cm^2^ were imported in Eclipse treatment planning system (Version 10.0, Varian Medical Systems, Palo Alto, CA) for the configuration of AXB. All data presented in this study were collected from a commissioned Varian Truebeam™ accelerator equipped with a Millennium 120 multileaf collimator (MLC, with spatial resolution of 5 and 10 mm for the central and outer 20 cm, respectively.

### CT scanning and contouring of organs at risk (OARs)

This retrospective study was approved by the ethics committee at the Cancer Hospital of Shantou University Medical College. Informed consent was obtained from all subjects prior to the study. During March 2012 and May 2013, 10 patients suffering from stage I NSCLC at our hospital were enrolled in this study. The CT datasets were acquired using a 16-slice CT scanner (The Philips Brilliance CT Big Bore Oncology Configuration, Cleveland, OH, USA) for all patients. Four dimensional computed tomography (4DCT) scanning and conventional enhanced 3D scanning with intravenous (IV) contrast was performed for 3 and 7 patients, respectively. Scanning was acquired at a 3 mm slice thickness for both 3D and 4DCT. CT images were then transferred to Eclipse treatment planning system. For patients with conventional enhanced scanning, gross tumor volume (GTV) was contoured by an experienced radiation oncologist under the CT pulmonary windows, and the planning target volume (PTV) was acquired according to the tumor motion under fluoroscopic examination with the aid of a simulator. For patients with 4DCT scanning, GTV accounting for tumor motion on all 10 phases of the 4DCT were contoured in the same way. These 10 phases of the GTV were then combined to form the internal target volume (ITV). To account for set-up uncertainties and potential baseline tumor shift, PTV was expanded with a uniform 5 mm margin from ITV. The PTV was then split into two substructures: PTV_soft, with a density in the soft-tissue range (0.590-0.985 g/cm^3^), and PTV_lung with a density in the lung range (0.011-0.590 g/cm^3^) [12]. All plans were carried out on the enhanced CT. OARs, including the aorta, bronchial tree, chest wall, spinal cord, esophagus, heart, ipsilateral and contralateral lung were contoured according to the RTOG 0915 report [20].

### Treatment planning

The prescription and dose constraints for the target and OARs in this study followed RTOG 0915 criteria [20]. Prescription was set to 4 fractions of 12 Gy. Treatment planning was designed with two volumetric modulated arc therapy (VMAT)-based partial arcs. Rotation angles were chosen to avoid the beams from entering the contralateral lung. Collimator angles for all plans were set to 30° in one arc and the complementary angle for another. The same beam arrangement and optimization constraints were employed for the 6XFFF and 10XFFF beams. Plans of different beam energy were optimized, selecting a maximum dose rate of 1400 MU/min and 2400 MU/min for 6XFFF and 10XFFF beams, respectively. Plan optimization was performed with the progressive resolution optimizer (PRO) algorithm implemented in Eclipse 10.0. Minimum and maximum dose constraints to the PTV were added during the planning optimization. Additionally, a dose-constraining structure (2 cm away from the PTV) was created to ensure a steep dose fall-off outside the PTV. The final dose calculation was normalized to guarantee that 95% of PTV received the prescribed dose. Dose calculations were carried out using AAA and AXB with a CGS of 2.5 mm and 1 mm, taking into account heterogeneity correction. For AXB calculation, we used the same monitor units (MU) and identical MLC settings as AAA algorithm and the “dose-to-medium” dose reporting mode was employed [12,21]. Accordingly, treatment plans of AAA with 2.5 mm and 1.0 mm CGS and AXB with 2.5 mm and 1.0 mm CGS were performed for each patient, respectively.

### Evaluation of the plans and statistical analysis

For the PTV, the evaluation parameters included the minimum, maximum and the mean dose. For the OARs, the analysis included the maximum, mean dose and a set of appropriate V_x_ values. V_x_ was the volume of the organ receiving a dose of x or more. For example, V_40_ was the volume of organ receiving a dose of 40 Gy or more. The absolute doses in this study were presented in Gy and all the data were rounded to the nearest tenth. To determine the difference between plans, Wilcoxon signed-rank tests were performed using SPSS 17.0 (Chicago, IL). Differences were considered statistically significant when *p* < 0.05.

## Results

**Figure 1:**
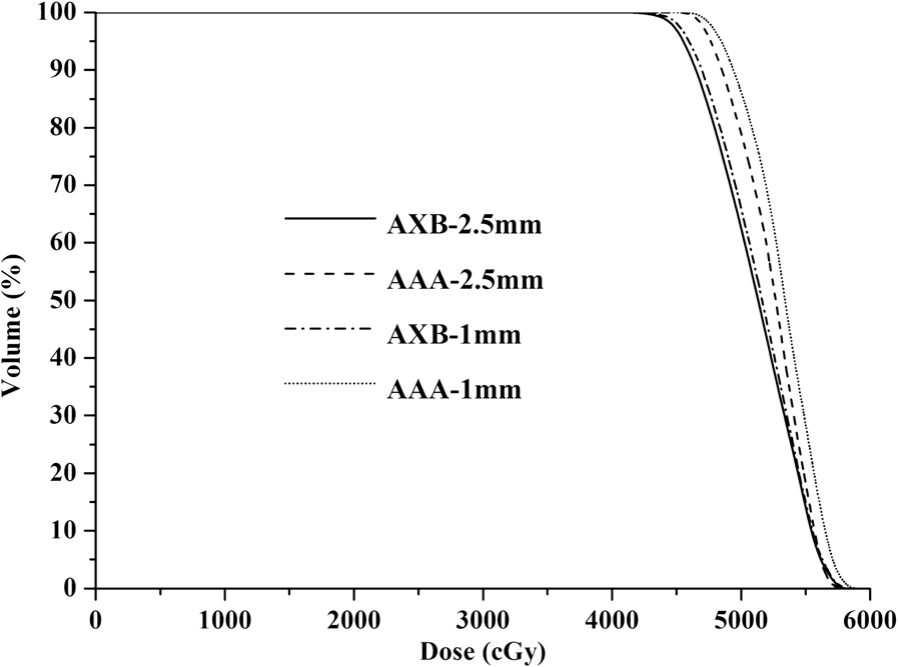
**DVH of PTV_lung calculated by different algorithms and CGS.**

**Figure 2:**
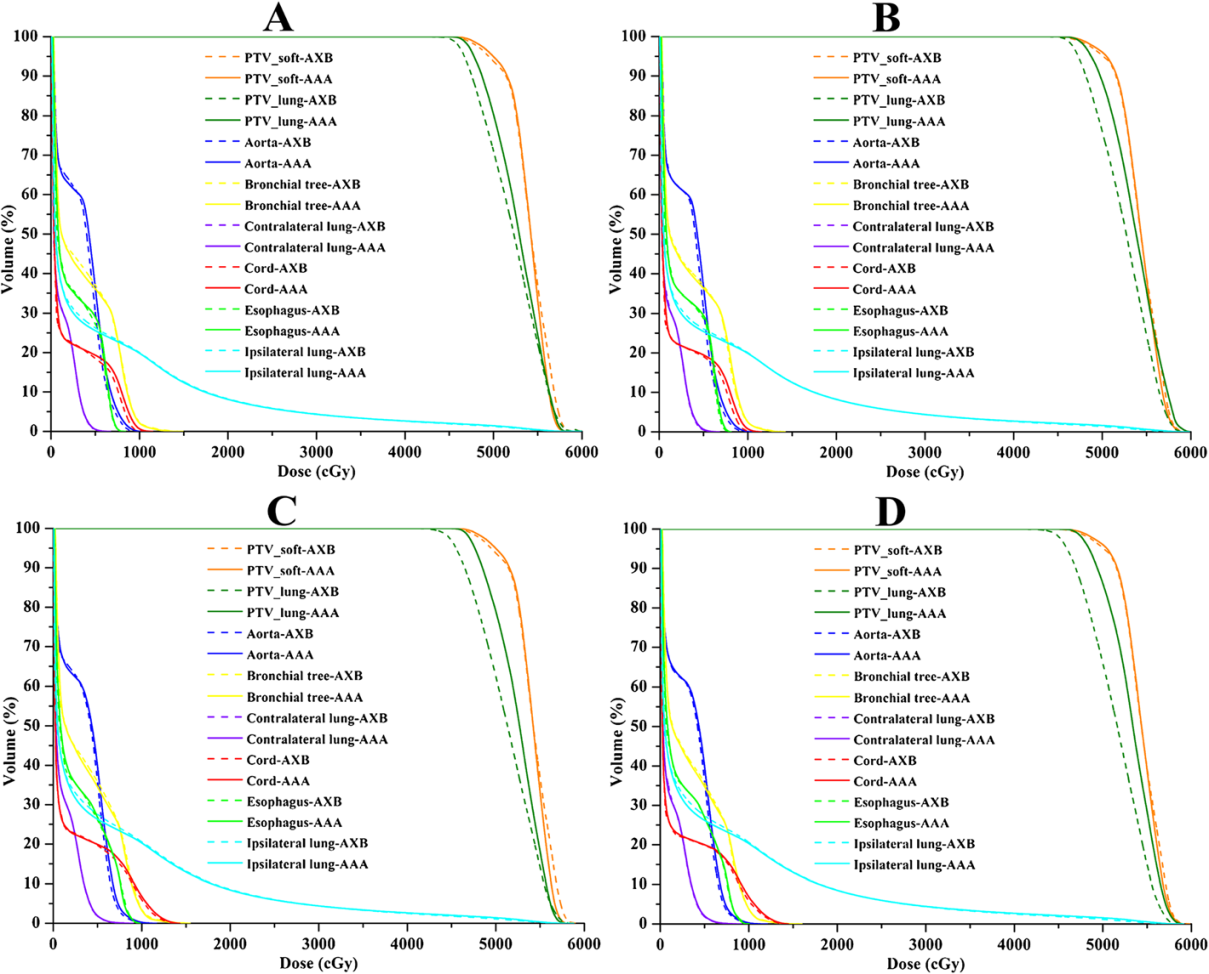
**Mean DVH of PTV and OARs. (A)** 6XFFF beam with 2.5 mm CGS, **(B)** 6XFFF beam with 1 mm CGS, **(C)** 10XFFF beam with 2.5 mm CGS and **(D)** 10XFFF beam with 1 mm CGS.

**Figure 3:**
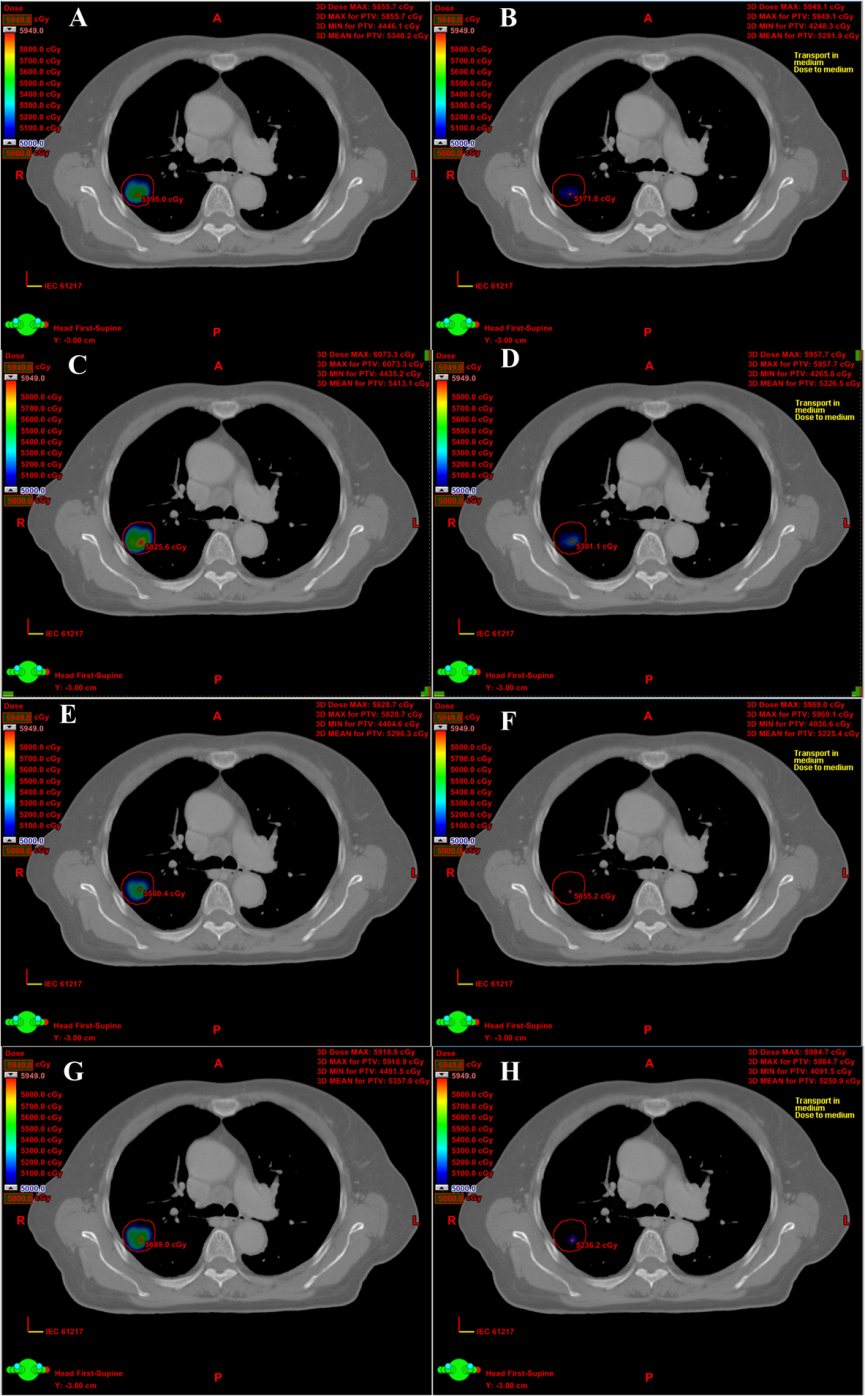
**Dose distributions of the two algorithms from one representative case. (A)** 6XFFF beam with AAA and 2.5 mm CGS, **(B)** 6XFFF beam with AXB and 2.5 mm CGS, **(C)** 6XFFF beam with AAA and 1 mm CGS, **(D)** 6XFFF beam with AXB and 1 mm CGS, **(E)** 10XFFF beam with AAA and 2.5 mm CGS, **(F)** 10XFFF beam with AXB and 2.5 mm CGS, **(G)** 10XFFF beam with AAA and 1 mm CGS, **(H)** 10XFFF beam with AXB and 1 mm CGS.

The dose difference between the AAA and AXB algorithm for SBRT plans of 10 lung cancer patients were summarized in Table 1 (6XFFF beam) and Table 2 (10XFFF beam). A considerable difference between the two algorithms was observed in PTV_lung, particularly when the 10XFFF beam plan with 1 mm CGS was used. A lower minimum dose and mean dose to PTV_lung were estimated by AXB than by AAA, regardless of whether 6XFFF or 10XFFF beam was used. Specifically, the dose directed to PTV_lung predicted by AXB was 1.33 ± 1.12% (6XFFF beam with 2.5 mm CGS) and 2.81 ± 2.33% (10XFFF beam with 2.5 mm CGS) lower compared with AAA. Furthermore, the dose difference between the two algorithms became larger by using 1 mm CGS (Figure 1). It was 2.33 ± 1.37% (6XFFF beam with 1 mm CGS) and 3.34 ± 1.76% (10XFFF beam with 1 mm CGS) lower compared with AAA. However, the minimum, maximum and mean dose directed to PTV_soft was comparable for the two algorithms, regardless of whether the 6XFFF or 10XFFF beam was used. When OARs were concerned, AXB always predicted a slightly lower dose than AAA. For example, AXB predicted a slightly lower dose to the aorta, chest wall, spinal cord and esophagus for the 6XFFF and 10XFFF beams. All of the dose change was statistically significant with *p* < 0.05. However, V_5_ and V_10_ of the ipsilateral lung were statistically higher with the AXB algorithm compared to the AAA. Figure 2 displayed the mean dose volume histograms (DVHs) of PTV and OARs at 6XFFF beam with 2.5 mm CGS, 6XFFF beam with 1 mm CGS, 10XFFF beam with 2.5 mm CGS and 10XFFF beam with 1 mm CGS, respectively. The dose distribution between the two algorithms from one representative case was illustrated in Figure 3.

**Table 1:** **Summary of the dose differences calculated by the AAA and AXB algorithm with 2.5 and 1 mm CGS for the 6XFFF beam**

**Structures**	**Metrics**	**Unit**	**AAA**_**2.5mm**_	**AXB**_**2.5mm**_	**AAA**_**1mm**_	**AXB**_**1mm**_
PTV_lung	D_min_	Gy	44.0 ± 0.8	42.0 ± 1.3^a^	44.6 ± 0.6	42.2 ± 2.0^b^
	D_max_	Gy	57.2 ± 1.7	57.6 ± 1.7	58.5 ± 1.5	58.0 ± 2.0
	D_mean_	Gy	51.9 ± 0.8	51.2 ± 1.2^a^	52.9 ± 0.8	51.6 ± 1.2^b^
PTV_soft	D_min_	Gy	46.9 ± 3.1	46.5 ± 3.3	46.6 ± 2.5	46.5 ± 3.0
	D_max_	Gy	57.2 ± 1.8	57.4 ± 2.1	58.1 ± 2.1	57.9 ± 1.9
	D_mean_	Gy	53.1 ± 1.8	52.9 ± 1.7	52.9 ± 2.0	52.9 ± 1.7
Aorta	D_max_	Gy	12.9 ± 9.7	12.6 ± 9.8^a^	12.9 ± 9.6	12.4 ± 9.3^b^
	D_mean_	Gy	2.6 ± 2.2	2.5 ± 2.1^a^	2.6 ± 2.2	2.5 ± 2.1^b^
Bronchial tree	D_max_	Gy	14.3 ± 10.4	14.4 ± 10.6	14.4 ± 10.4	14.4 ± 10.6
	D_mean_	Gy	3.5 ± 2.5	3.5 ± 2.5	3.6 ± 2.6	3.5 ± 2.5
Chest wall	V_45_	%	1.2 ± 1.9	1.1 ± 1.7	1.3 ± 1.9	1.2 ± 1.8^b^
	V_30_	%	7.3 ± 8.9	7.0 ± 8.4	7.4 ± 8.9	7.0 ± 8.4^b^
	V_20_	%	36.3 ± 31.2	34.1 ± 29.9^a^	37.2 ± 31.3	34.8 ± 30.1
	D_mean_	Gy	5.5 ± 2.4	5.4 ± 2.4^a^	5.5 ± 2.4	5.4 ± 2.4^b^
Spinal cord	D_max_	Gy	8.0 ± 2.9	7.5 ± 2.7^a^	8.2 ± 2.9	7.7 ± 2.8^b^
	D_mean_	Gy	1.5 ± 0.5	1.4 ± 0.4^a^	1.5 ± 0.5	1.4 ± 0.4^b^
Esophagus	D_max_	Gy	9.2 ± 3.4	8.9 ± 3.4^a^	9.3 ± 3.4	8.9 ± 3.4^b^
	D_mean_	Gy	2.0 ± 0.7	1.9 ± 0.7^a^	2.0 ± 0.7	1.9 ± 0.7^b^
Heart	D_max_	Gy	18.1 ± 10.5	17.8 ± 10.6	18.2 ± 10.4	18.0 ± 10.5
	D_mean_	Gy	1.4 ± 1.2	1.4 ± 1.2	1.4 ± 1.2	1.4 ± 1.2
Ipsilateral lung	V_5_	%	24.5 ± 7.9	25.1 ± 8.0^a^	24.4 ± 7.8	25.0 ± 8.0^b^
	V_10_	%	17.0 ± 7.6	17.1 ± 7.7	17.1 ± 7.6	17.1 ± 7.6
	V_20_	%	7.7 ± 4.7	7.6 ± 4.2	7.7 ± 4.2	7.6 ± 4.2
	D_mean_	Gy	5.0 ± 1.9	5.0 ± 2.0	5.0 ± 2.0	5.0 ± 2.0
Contralateral lung	V_5_	%	0.6 ± 0.9	0.6 ± 0.9	0.7 ± 0.9	0.6 ± 0.9^b^
	D_mean_	Gy	0.9 ± 0.4	0.9 ± 0.3	0.9 ± 0.4	0.9 ± 0.3

Abbreviations: PTV_lung = PTV with a density in the lung range; PTV_soft = PTV with a density in the soft-tissue range; Dmin = minimum dose; D_max_ = maximum dose; D_mean_ = mean dose; V_x_ was the volume of the organ receiving a dose of x or more; ^a^Wilcoxon signed-rank tests with *p* value <0.05 between AXB and AAA plan with 2.5 mm CGS; ^b^Wilcoxon signed-rank tests with *p* value <0.05 between AXB and AAA plan with 1 mm CGS.

**Table 2:** **Summary of the dose differences calculated by the AAA and AXB algorithm with 2.5 and 1 mm CGS for the 10XFFF beam**

**Structures**	**Metrics**	**Unit**	**AAA**_**2.5mm**_	**AXB**_**2.5mm**_	**AAA**_**1mm**_	**AXB**_**1mm**_
PTV_lung	D_min_	Gy	44.2 ± 0.7	39.9 ± 2.1^a^	44.5 ± 0.7	40.4 ± 2.9^b^
	D_max_	Gy	57.4 ± 1.2	57.8 ± 2.0	58.5 ± 0.9	58.3 ± 1.8
	D_mean_	Gy	51.9 ± 0.6	50.5 ± 1.6^a^	52.7 ± 0.6	50.9 ± 1.3^b^
PTV_soft	D_min_	Gy	47.6 ± 2.9	47.4 ± 3.0	47.4 ± 2.8	46.9 ± 2.6
	D_max_	Gy	57.1 ± 2.0	57.7 ± 2.4	57.8 ± 2.0	58.3 ± 2.2^b^
	D_mean_	Gy	53.5 ± 1.7	53.3 ± 1.8	53.5 ± 1.8	53.4 ± 1.8
Aorta	D_max_	Gy	13.7 ± 10.1	13.7 ± 10.2	13.8 ± 10.1	13.8 ± 10.0
	D_mean_	Gy	2.8 ± 2.5	2.7 ± 2.4^a^	2.8 ± 2.5	2.7 ± 2.4^b^
Bronchial tree	D_max_	Gy	14.8 ± 10.0	14.9 ± 10.1	15.0 ± 10.0	15.0 ± 10.0
	D_mean_	Gy	3.9 ± 2.8	3.9 ± 2.8	3.9 ± 2.8	3.9 ± 2.8
Chest wall	V_45_	%	1.3 ± 2.0	1.3 ± 2.0	1.4 ± 2.1	1.3 ± 2.0
	V_30_	%	8.1 ± 9.5	7.9 ± 9.1	8.2 ± 9.5	8.0 ± 9.2^b^
	V_20_	%	36.3 ± 30.6	35.1 ± 30.0^a^	37.1 ± 30.8	35.9 ± 30.2
	D_mean_	Gy	5.5 ± 2.4	5.4 ± 2.4^a^	5.5 ± 2.4	5.4 ± 2.4^b^
Spinal cord	D_max_	Gy	9.4 ± 3.8	9.1 ± 3.7^a^	9.5 ± 3.8	9.3 ± 3.7^b^
	D_mean_	Gy	1.6 ± 0.6	1.6 ± 0.5^a^	1.6 ± 0.6	1.6 ± 0.5^b^
Esophagus	D_max_	Gy	9.9 ± 3.6	9.8 ± 3.7	10.0 ± 3.5	10.0 ± 3.7
	D_mean_	Gy	2.1 ± 0.8	2.1 ± 0.8^a^	2.1 ± 0.8	2.1 ± 0.8^b^
Heart	D_max_	Gy	18.0 ± 10.1	18.2 ± 11.0	18.1 ± 10.8	18.3 ± 10.9
	D_mean_	Gy	1.5 ± 1.3	1.5 ± 1.3^a^	1.5 ± 1.3	1.5 ± 1.3^b^
Ipsilateral lung	V_5_	%	25.4 ± 8.1	26.5 ± 8.3^a^	25.2 ± 8.1	26.4 ± 8.3^b^
	V_10_	%	17.7 ± 7.8	18.0 ± 8.0^a^	17.7 ± 7.8	18.0 ± 8.0^b^
	V_20_	%	7.9 ± 4.4	8.0 ± 4.5	8.0 ± 4.5	8.0 ± 4.5
	D_mean_	Gy	5.2 ± 2.0	5.2 ± 2.0^a^	5.2 ± 2.0	5.2 ± 2.1
Contralateral lung	V_5_	%	1.6 ± 1.4	1.5 ± 1.4	1.7 ± 1.5	1.6 ± 1.5^b^
	D_mean_	Gy	1.0 ± 0.4	1.0 ± 0.4	1.0 ± 0.4	1.0 ± 0.4

Abbreviations: PTV_lung = PTV with a density in the lung range; PTV_soft = PTV with a density in the soft-tissue range; Dmin = minimum dose; D_max_ = maximum dose; D_mean_ = mean dose; V_x_ was the volume of the organ receiving a dose of x or more; ^a^Wilcoxon signed-rank tests with *p* value <0.05 between AXB and AAA plan with 2.5 mm CGS; ^b^Wilcoxon signed-rank tests with *p* value <0.05 between AXB and AAA plan with 1 mm CGS.

## Discussion

The International Commission on Radiation Units (ICRU) recommended an overall accuracy of 5% in the delivery of absorbed dose [22], and the American Association of Physicists in Medicine (AAPM) advised that the uncertainty in the computed dose distribution should be less than 2% [23]. Both of the AXB and AAA algorithms exhibit similar dose prediction in homogeneous media. However, AXB was reported to be more accurate in heterogeneous medium [2,5-7]. Accordingly, it is essential for us to accurately determine the dose delivered to lung tumor in which the water density target is always surrounded by low density lung tissue. For lung radiotherapy treatment, PTV in the low density range is originated either from the tumor motion uncertainty [24] or uncertainty in patient positioning and alignment of the therapeutic beams during the treatment [25]. We determined that the mean dose of PTV_lung predicted by the AXB algorithm was up to 3.34% lower compared to the AAA by using the 10XFFF beam energy. Although the dose to PTV_lung was reduced, the PTV_soft dose was not affected. We acknowledged this as it was an interesting finding. For lung SBRT treatment, the PTV_lung dose changes were of positive significance because they could be associated with significant changes in tumor control probability (TCP) and normal tissue complication probabilities (NTCP) according to the AAPM Report No. 85 [23]. Therefore, determination of the dosimetric effect with AXB on TCP will be an interesting work for our future study.

The dosimetric impact of the AXB algorithm has been investigated in several studies. Fogliata *et al.* [12] compared the AXB to AAA in patients with stage III NSCLC and concluded that a lower mean dose to soft tissue and a slightly higher mean dose in low-density lung tissue were obtained by the 6 MV beam with the AXB algorithm. Kan *et al.* [4] addressed this issue using IMRT and RapidArc techniques in nasopharyngeal carcinoma patients and found that AXB presented a 1% higher dose in air compared with AAA. The same mean target dose between the two algorithms was demonstrated by Liu *et al.* [15] in SBRT treatment for NSCLC patients. Kroon *et al.* [16] reported that D_98%_ of PTV was lower using AXB, whereas the mean dose of PTV was comparable for the lung SBRT treatment plan. Our finding indicating that the mean dose of PTV_lung was obviously lower with the AXB algorithm differs from the results of these studies. These contradictions are reasonable because the differences in lung doses calculated by AAA and AXB were influenced by many factors, such as beam energy, field size, field number and the density of lung tissue [4,6]. Another possibility that may lead to the inconsistency was the use of FFF beam in our research. Unlike flattened beams, FFF ones can provide softer energy and a cone-like profile. Moreover, some differences in the electron contamination parameters were acquired by removal of the flattening filter, which was not observed in the flattened beams [26]. These differences may influence the dose differences between the two algorithms and further investigation is necessary.

Additionally, we also determined that AXB estimated a lower dose for most of the OARs, whereas V_5_ and V_10_ of the ipsilateral lung were significantly higher with AXB. The higher dose prediction in ipsilateral lung could be explained by the shape of the profiles obtained by the AXB algorithm. Kan *et al.* [4] reported that the dose profile in air indicated a lower dose inside the field but a higher out-of-field dose predicted by AXB. When the gantry rotated around the patient, all OARs were inside the treatment field at some degrees of gantry rotation during the entire treatment process except for the ipsilateral lung. This helped to explain why V_5_ and V_10_ of the ipsilateral lung increased with the AXB algorithm. V_5_ was reported to be a dosimetric factor that was predictive of radiation-induced pneumonitis (RIP) [27], however, as the dose difference was minor (0.6-1.6% higher) with the AXB and its clinical effect could be neglected.

Our finding that the CGS also contributed to the dose difference between AAA and AXB is interesting. CGS was reported to be associated with dose estimation and calculation accuracy. Chung *et al.* [13] and Mittauer *et al.* [14] found that CGS influenced dose estimation in head and neck treatment. Ong *et al.* [28] found that 1.0 mm CGS improved the accuracy of dose calculation compared with 2.5 mm for the AAA algorithm. Kan *et al.* [9] also reported that AXB with 1.0 mm CGS resulted in a significant improvement in the dose accuracy (within 3%). Smaller grid resolution reduces the averaging effect and results in better sampling of the structure voxels to the calculation grid [9]. Interestingly, our result demonstrated that CGS also influenced the relative dose difference between the AAA and AXB algorithm. By using 1 mm CGS, dose difference in PTV_lung between the two algorithms was greater compared with 2.5 mm CGS, regardless of whether the 6XFFF beam or 10XFFF beam was used. That’s due to the ability of AAA and AXB algorithms to adapt the CGS to ensure the dose accuracy in the axis perpendicular to the image slices. If the slice thickness is larger than the defined grid size, AAA and AXB may calculate the dose on dose planes between the image slices. And if the slice spacing is smaller than the defined grid size, AAA and AXB may skip calculating the dose on some slices. Based on the fact that the AXB algorithm predicts lower dose in each image slice, the dose difference between the two algorithms will undoubtedly become larger with 1 mm CGS because it involves more image slices in dose prediction. Compared with previous research, our result can more truly reflect the dosimetric difference between the two algorithms with 1 mm CGS due to its better calculation accuracy than 2.5 mm CGS. This was another factor that led to the inconsistency with other previous studies. To our knowledge, the potential impact of CGS on the dose difference between AAA and AXB has not been reported and analyzed and this study is the first to address this issue.

Our study has certain limitations. Although some recent publications have demonstrated the accuracy of AXB algorithm, few studies have verified the dosimetric performance of AXB algorithm in a SBRT plan produced from FFF beams. Our further work will focus on lung SBRT plans where small fields and air cavity are involved. Additionally, Kan *et al.* [9] recommended the use of 1 mm CGS instead of 2.5 mm whenever possible for stereotactic plans using a slice spacing of 1.25 mm, particularly when PTV contains relatively large areas of low density tissue. Whether different slice spacing will influence the relative dose difference between the two algorithms will be another area of interest for us.

## Conclusions

The use of AXB mainly presents a lower dose in PTV_lung compared to the dose that is calculated from AAA in SBRT treatment for lung cancer, regardless of whether the 6XFFF or 10XFFF beam is used. The dose differences become larger by using higher beam quality and smaller CGS.

## Abbreviations

AXB: Acuros XB
AAA: Anisotropic Analytical Algorithm
SBRT: Stereotactic body radiotherapy
CGS: Calculation grid size
FFF: Flattening filter free
MU: Monitor unit
MLC: Multileaf collimator
PTV: Planning target volume
GTV: Gross tumor volume
ITV: Internal target volume
OARs: Organs at risk
MC: Monte Carlo
NSCLC: Non-small cell lung cancer
4DCT: 4 dimensional computed tomography
RTOG: Radiation Therapy Oncology Group
PRO: Progressive resolution optimizer
DVH: Dose volume histogram
ICRU: International Commission on Radiation Units & Measurements
AAPM: American Association of Physicists in Medicine
TCP: Tumor control probability
NTCP: Normal tissue complication probabilities
RIP: Radiation-induced pneumonitis

## References

[1] Ono K, Endo S, Tanaka K, Hoshi M, Hirokawa Y (2010). Dosimetric verification of the anisotropicanalytical algorithm in lung equivalent heterogeneities with and without bone equivalent heterogeneities. Med Phys.

[2] Han T, Followill D, Mikell J, Repchak R, Molineu A, Howell R (2013). Dosimetric impact of Acuros XB deterministic radiation transport algorithm for heterogeneous dose calculation in lung cancer. Med Phys.

[3] Kan MW, Cheung JY, Leung LH, Lau BM, Yu PK (2011). The accuracy of dose calculations by anisotropic analytical algorithms for stereotactic radiotherapy in nasopharyngeal carcinoma. Phys Med Biol.

[4] Kan MW, Leung LH, Yu PK (2013). Dosimetric impact of using the Acuros XB algorithm for intensity modulated radiation therapy and RapidArc planning in nasopharyngeal carcinomas. Int J Radiat Oncol Biol Phys.

[5] Bush K, Gagne IM, Zavgorodni S, Ansbacher W, Beckham W (2011). Dosimetric validation ofAcuros XB with Monte Carlo methods for photon dose calculations. Med Phys.

[6] Fogliata A, Nicolini G, Clivio A, Vanetti E, Cozzi L (2011). Dosimetric evaluation of Acuros XB Advanced Dose Calculation algorithm in heterogeneous media. Radiat Oncol.

[7] Han T, Mikell JK, Salehpour M, Mourtada F (2011). Dosimetric comparison of Acuros XB deterministic radiation transport method with Monte Carlo and model-based convolution methods in heterogeneous media. Med Phys.

[8] Han T, Mourtada F, Kisling K, Mikell J, Followill D, Howell R (2012). Experimental validation of deterministic Acuros XB algorithm for IMRT and VMAT dose calculations with the Radiological Physics Center's head and neck phantom. Med Phys.

[9] Kan MW, Leung LH, Yu PK (2012). Verification and dosimetric impact of Acuros XB algorithm on intensity modulated stereotactic radiotherapy for locally persistent nasopharyngeal carcinoma. Med Phys.

[10] Hoffmann L, Jorgensen MB, Muren LP, Petersen JB (2012). Clinical validation of the Acuros XB photon dose calculation algorithm, a grid-based Boltzmann equation solver. Acta Oncol.

[11] Fogliata A, Nicolini G, Clivio A, Vanetti E, Cozzi L (2011). On the dosimetric impact of inhomogeneity management in the Acuros XB algorithm for breast treatment. Radiat Oncol.

[12] Fogliata A, Nicolini G, Clivio A, Vanetti E, Cozzi L (2012). Critical appraisal of Acuros XB and Anisotropic Analytic Algorithm dose calculation in advanced non-small-cell lung cancer treatments. Int J Radiat Oncol Biol Phys.

[13] Chung H, Jin H, Palta J, Suh TS, Kim S (2006). Dose variations with varying calculation grid size in head and neck IMRT. Phys Med Biol.

[14] Mittauer K, Lu B, Yan G, Kahler D, Gopal A, Amdur R (2013). A study of IMRT planning parameters on planning efficiency, delivery efficiency, and plan quality. Med Phys.

[15] HW L i, Nugent Z, Clayton R, Dunscombe P, Lau H, Khan R (2013). Clinical impact of using the deterministic patient dose calculation algorithm Acuros XB for lung stereotactic body radiation therapy. Acta Onco.

[16] Kroon PS, Hol S, Essers M (2013). Dosimetric accuracy and clinical quality of Acuros XB and AAA dose calculation algorithm for stereotactic and conventional lung volumetric modulated arc therapy plans. Radiat Oncol.

[17] Ong CL, Verbakel WF, Dahele M, Cuijpers JP, Slotman BJ, Senan S (2012). Fast arc delivery for stereotactic body radiotherapy of vertebral and lung tumors. Int J Radiat Oncol Biol Phys.

[18] Lang S, Shrestha B, Graydon S, Cavelaars F, Linsenmeier C, Hrbacek J (2013). Clinical application of flattening filter free beams for extracranial stereotactic radiotherapy. Radiother Oncol.

[19] Vassiliev ON, Titt U, Ponisch F, Kry SF, Mohan R, Gillin MT (2006). Dosimetric properties of photon beams from a flattening filter free clinical accelerator. Phys Med Biol.

[20] Radiation Therapy Oncology Group. A Randomized Phase II Study Comparing 2 Stereotactic Body Radiation Therapy (SBRT) Schedules for Medically Inoperable Patients with Stage I Peripheral Non-Small Cell Lung Cancer. 2014. [http://www.rtog.org/ClinicalTrials/ProtocolTable/StudyDetails.aspx?action=openFile&FileID=4673]. Accessed 12 Jan 2015.

[21] Ma CM, Li J (2011). Dose specification for radiation therapy: dose to water or dose to medium?. Phys Med Biol.

[22] International Commission on Radiation Units & Measurements. Determination of absorbed dose in a patient irradiated by beams of X or gamma rays in radiotherapy procedures. ICRU Report 24; 1976.

[23] American Asociation of Physicists in Medicine. Tissue inhomogeneity corrections for megavoltage photon beams. 2004. https://www.aapm.org/pubs/reports/rpt_85.pdf. Accessed 12 Jan 2015.

[24] Ezhil M, Vedam S, Balter P, Choi B, Mirkovic D, Starkschall G (2009). Determination of patient-specific internal gross tumor volumes for lung cancer using four-dimensional computed tomography. Radiat Oncol.

[25] International Commission on Radiation Units & Measurements (2007). Prescribing, recording, and reporting photon-beam therapy. J ICRU.

[26] Fogliata A, Nicolini G, Clivio A, Vanetti E, Mancosu P, Cozzi L (2011). Dosimetric validation of the Acuros XB Advanced Dose Calculation algorithm: fundamental characterization in water. Phys Med Biol.

[27] Baker R, Han G, Sarangkasiri S, DeMarco M, Turke C, Stevens CW (2013). Clinical and dosimetric predictors of radiation pneumonitis in a large series of patients treated with stereotactic body radiation therapy to the lung. Int J Radiat Oncol Biol Phys.

[28] Ong CL, Cuijpers JP, Senan S, Slotman BJ, Verbakel WF (2011). Impact of the calculation resolution of AAA for small fields and RapidArc treatment plans. Med Phys.

